# Addressing the Analytic Challenges of Cross-Sectional Pediatric Pneumonia Etiology Data

**DOI:** 10.1093/cid/cix147

**Published:** 2017-05-29

**Authors:** Laura L. Hammitt, Daniel R. Feikin, J. Anthony G. Scott, Scott L. Zeger, David R. Murdoch, Katherine L. O’Brien, Maria Deloria Knoll

**Affiliations:** 1 Department of International Health, International Vaccine Access Center, Johns Hopkins Bloomberg School of Public Health, Baltimore, Maryland;; 2 Kenya Medical Research Institute–Wellcome Trust Research Programme, Kilifi;; 3 Division of Viral Diseases, National Center for Immunizations and Respiratory Diseases, Centers for Disease Control and Prevention, Atlanta, Georgia;; 4 Department of Infectious Disease Epidemiology, London School of Hygiene & Tropical Medicine, United Kingdom;; 5 Department of Biostatistics, Johns Hopkins Bloomberg School of Public Health, Baltimore, Maryland; and; 6 Department of Pathology, University of Otago, and; 7 Microbiology Unit, Canterbury Health Laboratories, Christchurch, New Zealand

**Keywords:** pneumonia, etiology, case-control analysis, attributable fraction analysis, latent class analysis.

## Abstract

Despite tremendous advances in diagnostic laboratory technology, identifying the pathogen(s) causing pneumonia remains challenging because the infected lung tissue cannot usually be sampled for testing. Consequently, to obtain information about pneumonia etiology, clinicians and researchers test specimens distant to the site of infection. These tests may lack sensitivity (eg, blood culture, which is only positive in a small proportion of children with pneumonia) and/or specificity (eg, detection of pathogens in upper respiratory tract specimens, which may indicate asymptomatic carriage or a less severe syndrome, such as upper respiratory infection). While highly sensitive nucleic acid detection methods and testing of multiple specimens improve sensitivity, multiple pathogens are often detected and this adds complexity to the interpretation as the etiologic significance of results may be unclear (ie, the pneumonia may be caused by none, one, some, or all of the pathogens detected). Some of these challenges can be addressed by adjusting positivity rates to account for poor sensitivity or incorporating test results from controls without pneumonia to account for poor specificity. However, no classical analytic methods can account for measurement error (ie, sensitivity and specificity) for multiple specimen types and integrate the results of measurements for multiple pathogens to produce an accurate understanding of etiology. We describe the major analytic challenges in determining pneumonia etiology and review how the common analytical approaches (eg, descriptive, case-control, attributable fraction, latent class analysis) address some but not all challenges. We demonstrate how these limitations necessitate a new, integrated analytical approach to pneumonia etiology data.

*Many an object is not seen, though it falls within the range of our visual ray, because it does not come within the range of our intellectual ray, i.e. we are not looking for it. So, in the largest sense, we find only the world we look for.*

—Henry David Thoreau, *Journal*, 2 July 1857

Investigation of the etiology of childhood pneumonia has long guided targeted prevention and treatment strategies. As the diseased tissue (ie, the lung) is not routinely sampled for testing [1], pneumonia etiology is typically inferred using test results of specimens from other anatomical sites. In the traditional approach to pneumonia etiology, detection of pathogens has been synonymous with causation, without fully accounting for the limitations of the testing methods.

For tests with low sensitivity (eg, blood culture for bacteria), researchers can accept these imperfect results as “conservative estimates” of a pathogen’s role. Tests with exquisite analytical sensitivity (ie, ability to detect presence of a pathogen in a specimen) will detect a pathogen in the upper respiratory tract of the vast majority of children with pneumonia and many will test positive for >1 pathogen. However, use of an increasing number of imperfectly specific tests results in a trade-off between diagnostic yield and diagnostic accuracy and adds complexity when combining and interpreting the results of these tests.

With the goal of improving the way we use available data to understand the etiology of pneumonia, we describe key analytic challenges and the existing analytic approaches that only partially address them (Table 1), and illustrate how the approach to analysis influences etiologic inferences.

**Table 1.** Limitations of Available Analytic Approaches to Address Challenges in Interpreting Data From Cross-Sectional Pneumonia Etiology Studies

Challenge	Possible Approaches	Limitation of Approaches
Imperfect diagnostic sensitivity of assays	1. Assume 100%	• Underestimates role of pathogen if true sensitivity is <100%
	2. Adjust for test sensitivity	• Adjustment will increase the number of positives, but it is not possible to attribute etiology to specific individuals • Not possible to integrate adjusted results at an individual level with other results
Imperfect diagnostic specificity of assays	1. Assume 100%	• Overestimates role of pathogen if true specificity is <100%
	2. Case-control odds ratio	• Cannot be applied to assays not available from controls • For measurements associated with case status, assumes specificity of measurement in cases is 100% • Researchers must set rules for interpretation (eg, whether a pathogen will be deemed to be causal if the odds ratio is >1 or only if statistically significantly >1). Statistical significance of findings (ie, assessment of causal association) is dependent on size of study • Odds ratio cannot be converted into a positive predictive value for a given individual
	3. Attributable fraction	• See points 1–3 under Case-control approach • Not possible to estimate results for a given individual or to combine results for multiple pathogens
	4. Quantification of pathogen load	• No diagnostic threshold established for most pathogens
Estimating etiology for pathogens not tested for	1. Ascribe “unknown” etiology to cases negative for all tested pathogens	• Underestimated if specificity of pathogens tested for is <100% (unless accounting for this using an attributable fraction approach) • Overestimated if sensitivity of pathogens tested for is <100%
Combining multiple test results^a^	1. Expert adjudication	• Time-intensive to deliberate on each case to assign etiology • Does not allow for quantification of uncertainty • Preconceptions of experts may bias results • Does not account for imperfect sensitivity • Process results in an all-or-nothing decision about each pathogen(s)
	2. Latent class analysis	• Cannot incorporate control data • Only feasible if the number of pathogens (classes) is constrained to a small number • The researcher must “interpret” the measurement distribution profiles of each class and ascribe an etiology

^a^This can include performing >1 test for a given pathogen or from different pathogens identified in different specimens.

## Challenge 1: Imperfect Diagnostic Sensitivity of Assays

Historically, pediatric pneumonia etiology studies usually only described clinical and microbiological findings in a series of cases. In studies relying on culture of sterile-site specimens (eg, blood, pleural fluid), pneumonia is attributed to the cultured pathogen. However, the sensitivity of blood culture is poor for detecting bacterial pneumonia, both because bacteremia in true cases of bacterial pneumonia is uncommon or intermittent and because of antibiotic effects [2].

### Approach to Challenge 1: Adjust for Test Diagnostic Sensitivity

**Figure 1.**
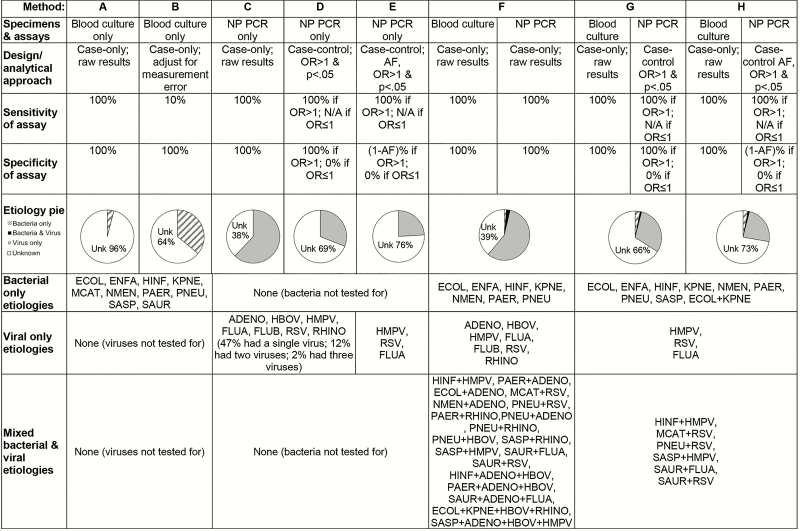
Differences in pneumonia etiology by specimens, assays, and analytical approaches. Pneumonia etiology using results of blood culture testing (*A*); results of blood culture adjusted for sensitivity (*B*); results of nasopharyngeal (NP) polymerase chain reaction (PCR) for 7 viruses (*C*); results of NP PCR, limited to pathogens for which the case-control odds ratio was significantly greater than 1 (type 1 error = 0.05) (*D*); results of method D after applying attributable fraction (AF) adjustment (*E*); results of blood culture and NP PCR from cases only (*F*); results of blood culture and NP PCR, limited to pathogens for which the NP PCR case-control odds ratio was significantly greater than 1 (type 1 error = 0.05) (*G*); results of method G after applying AF adjustment (*H*). Each “method” is a combination of the study design (case-only or case-control), analytical approach (raw results, adjustment for measurement error, odds ratio, AF), and assumed measurement error (ie, sensitivity and specificity) of the assay. The analyses were performed on a hypothetical study of 1000 pneumonia cases and 1000 controls (for case-control comparisons). The hatched slice represents a bacterial etiology (ie, cases positive by blood culture only); the black slice represents those with a mixed bacterial and viral etiology (ie, cases positive by blood culture and viral PCR); the solid gray slice represents viral etiology (ie, cases positive by viral PCR only). Abbreviations: ADENO, adenovirus; AF, attributable fraction; ECOL, *Escherichia coli*; ENFA, *Enterococcus faecium*; FLUA, influenza A; FLUB, influenza B; HBOV, human bocavirus; HINF, *Haemophilus influenzae*; HMPV, human metapneumovirus A/B; KPNE, *Klebsiella pneumoniae*; MCAT, *Moraxella catarrhalis*; N/A, not applicable; NMEN, *Neisseria meningitidis*; NP, nasopharyngeal; OR, odds ratio; PAER, *Pseudomonas aeruginosa*; PCR, polymerase chain reaction; PNEU, *Streptococcus pneumoniae*; RHINO, human rhinovirus; RSV, respiratory syncytial virus A/B; SASP, *Salmonella* species; SAUR, *Staphylococcus aureus*; Unk, unknown.

We can account for the poor sensitivity of blood culture for detection of pneumonia etiology by multiplying the proportion positive by the reciprocal of the sensitivity. For example, if 36 of 1000 (3.6%) children had positive blood cultures (Figure 1, method A), adjusting for a sensitivity of 10% would imply that 360 (36%) cases are attributable to those identified bacteria (Figure 1, method B). However, there is no information to guide which specific 324 additional children (360 assumed positive minus 36 test positive) with “false negative” blood cultures should be attributed to a bacterial etiology following the sensitivity adjustment since the sensitivity adjustment can be applied at the population level but not to individual patients. Another challenge is obtaining an accurate estimate of the sensitivity to use for this adjustment. For some bacterial species (eg, pneumococcus, *Haemophilus influenzae* type b), we can estimate the sensitivity of blood culture directly using lung aspirate studies or indirectly using vaccine probe studies by comparing the incidence of bacteremic pneumonia prevented relative to the incidence of all clinical pneumonia prevented. Unfortunately, empirical data are lacking for most bacterial species, which do not have effective vaccines to be used as probes, and sensitivity can also vary depending on antibiotic exposure prior to specimen collection.

## Challenge 2: Imperfect Diagnostic Specificity of Assays

Although nucleic acid detection tests (eg, polymerase chain reaction [PCR]) have very high specificity for the detection of respiratory pathogens in the *specimen*, these measurements have imperfect *diagnostic* specificity for determining the etiology of pneumonia. A respiratory virus can be detected in the nasopharynx of 25%–80% of children hospitalized with pneumonia, but many of these respiratory viruses are detected nearly as frequently in healthy children or children with upper respiratory tract infection but without pneumonia, making the etiologic significance of the results unclear [3–10]. Even the diagnostic specificity of tests of sterile-site specimens is imperfect. For example, presence of gram-negative rods in the blood of pneumonia cases with malnutrition and sepsis may not indicate the cause of the pneumonia, but merely that the integrity of the bowel wall is compromised in severely ill children. Furthermore, evidence indicates that the healthy lung is home to a diverse community of microbes and that pneumonia develops within this ecosystem [11]. Therefore, detection of organisms in lung tissue, long thought to be the gold standard assessment of pneumonia etiology, also has imperfect specificity.

**Figure 2.**
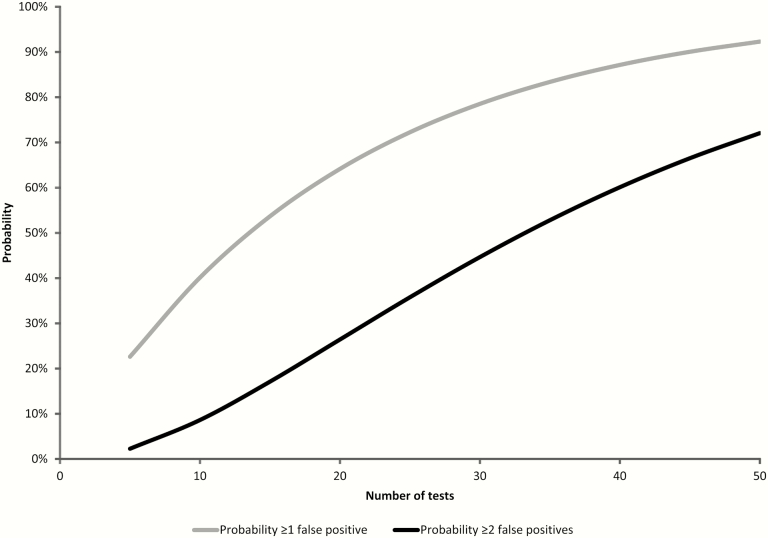
Probability of 1 or more false-positive test results with increasing number of tests performed. Probability calculation based on binomial theorem [eg, probability ≥1 false positive = 1 – probability of zero false positives = 1– (specificity^number of tests^)]. In this example, specificity is set at 95%.

As additional specimens and tests are incorporated into the diagnostic evaluation of children with pneumonia, the proportion in whom a potential pathogen is identified increases, but so does the proportion with false-positive results (Figure 2). For example, if independent PCR assays for 30 different pathogens are undertaken, each with a specificity of 95%, a false-positive result is expected in 79% of cases, and multiple false positives in 45% of cases.

### Approach to Challenge 2. A: Case-Control Odds Ratio Analysis

One approach to assess the role of detected pathogens in pneumonia cases is to compare their infection status (ie, laboratory test results) to people without pneumonia (ie, controls). The odds ratio (OR) describes the association between test results and pneumonia status. Conventionally, the OR is used to make a binary decision about a pathogen’s role: pathogens with ORs statistically significantly greater than 1 are considered associated with case status and cases positive for these pathogens have their pneumonia etiology attributed to them, whereas pathogens not meeting this criterion are excluded as potential causes of pneumonia. This approach assumes 100% specificity among cases who test positive for a pathogen (ie, pneumonia is attributed to that pathogen if it is detected in a case, despite the fact that it may have also been detected in some controls). The magnitude of the OR does not have interpretive value about the fraction of the etiologic contribution to pneumonia, nor about the positive predictive value of a test in a given individual. Additionally, the OR approach cannot be applied to measurements not available from controls (eg, induced sputum, lung aspirate, pleural fluid).

The use of control data in the assessment of pneumonia etiology is illustrated by a recent Swedish study that assessed 15 viral pathogens by PCR in nasopharyngeal (NP) specimens [9]. At least 1 virus was detected in 77% of 93 cases with radiographic pneumonia. However, 56% of 220 matched controls also had viruses detected. In the adjusted OR analysis, only 3 viruses (influenza, human metapneumovirus [HMPV], and respiratory syncytial virus [RSV]) were statistically significantly positively associated with case status; the other pathogens assessed (eg, bocavirus, parainfluenza virus) were not associated or were negatively associated with case status and therefore no etiology was attributed to them.

Having an OR not significantly greater than 1 does not rule out the pathogen as a cause of pneumonia, however (ie, absence of evidence of an association is not equivalent to evidence of absence of an association). For example, pneumococcus commonly colonizes the nasopharynx of children with and without pneumonia; detection of this pathogen in the upper respiratory tract of controls at a similar or higher prevalence compared with cases does not mean pneumococcus is not a cause of pneumonia. An OR <1 may arise when the specimen being tested has poor specificity for detecting the cause of pneumonia, or it may reflect bias (eg, a consequence of antibiotics reducing the prevalence of bacteria among cases). Additionally, interaction between potential pathogens may create a situation in which a pathogen sometimes appears to have a protective effect (eg, by competitively inhibiting the growth of another causally relevant pathogen).

Accepting the analytic and interpretive constraints of the OR approach, there are also important design considerations for case-control studies. Perhaps most important is the selection of the control group [12, 13]; if it excludes certain segments of the population that give rise to cases, results are prone to bias. Sample size is another important consideration as small studies will be biased toward detecting significant associations only for very prevalent pathogens or those most strongly associated with case status.

### Approach to Challenge 2. B: Attributable Fraction Analysis

The attributable fraction (AF) method addresses a limitation of the case-control OR approach—the assumption of 100% specificity among cases who test positive for a pathogen—by accounting for the pathogen prevalence among controls. Although initially developed as a way to assess the fraction of disease attributed to *exposure(s*) in noncommunicable disease epidemiology, recently this method has been used to attribute *etiology* [7, 14, 15]. The proportion of cases infected with a given pathogen for whom that pathogen is deemed responsible for their illness, ie, the “attributable fraction among the exposed” (AFE), is calculated as 1 – (1 / OR), where OR is the case-control odds ratio for that pathogen. Bruzzi et al proposed an extension of this method using multiple logistic regression to estimate the summary AF for multiple risk factors [16]; this approach has been applied in the field of diarrheal etiology to estimate the AF for a pathogen adjusted for other pathogens that are present [17].

Table 2 illustrates the calculation of the AFE and AF using case and control pathogen prevalences. For example, HMPV was detected in 8.2% of cases and 4.3% of controls, which yields an OR = 1.98 and AFE = 1 – (1 / 1.98) = 0.49 (ie, 49% of cases testing positive for HMPV are attributable to HMPV). The population AF for HMPV (ie, the proportion of all cases attributable to HMPV) is 4.1% (calculated as 49% AFE × 8.2% HMPV positive among cases).

**Table 2.** Calculation of the Attributable Fraction, the Fraction of Cases Attributed to Each of 7 Pathogens, in a Hypothetical Study of 1000 Cases With Pneumonia and 1000 Community Controls by Comparing Prevalence of Polymerase Chain Reaction Positivity in Nasopharyngeal Specimens

Pathogen	Prevalence in Cases, %	Prevalence in Controls, %	OR (95% CI)	AFE, %	AF, %
Adenovirus	11.0	12.7	0.85 (.65–1.12)	NA	NA
Human bocavirus	14.8	14.7	1.01 (.79–1.30)	1.3	0.2
HMPV	8.2	4.3	**1.98 (1.35**–**2.91**)	**49.5**	**4.1**
Influenza A	3.1	0.9	**3.40 (1.60**–**7.19**)	**70.6**	**2.2**
Influenza B	1.3	0.6	2.18 (.82–5.75)	54.1	0.7
RSV	21.4	2.4	**11.20 (7.21**–**17.41**)	**91.1**	**19.5**
Human rhinovirus	20.8	20.5	1.02 (.82–1.27)	2.1	0.4
Any virus	61.8	43.9			27.1

Bolded numbers are statistically significant.

Abbreviations: AF, attributable fraction calculated as prevalence in cases × AFE; AFE, attributable fraction among the exposed calculated as 1 – 1 / odds ratio; CI, confidence interval; HMPV, human metapneumovirus A/B; NA, not applicable; OR, odds ratio; RSV, respiratory syncytial virus A/B.

The sum of all pathogen-specific AFs is not constrained to 100%. As applied to risk factors, this means disease could have been prevented if any one of multiple causal exposures had been averted. When applied to infectious causes of pneumonia, if AFs sum to >100%, it is not clear if this indicates that pneumonia is definitely caused by >1 pathogen or reflects bias and confounding in estimating the OR. If AFs sum to <100% it may be because the sensitivity of the tests is usually not accounted for (ie, 100% sensitivity is assumed).

Although the AF approach improves on the OR approach by accounting for background (ie, control) prevalence, it has all of the other constraints of the OR approach: Pathogens with ORs not statistically significantly greater than 1 are excluded; sample size and pathogen prevalence influence which pathogens are statistically significant; it only applies to analyses involving a single measurement type for each pathogen; and it only applies to specimens collected from both cases and controls. Most pneumonia etiology studies violate these constraints; however, the AF approach is useful for other syndromes such as diarrhea where a single specimen type (eg, stool) can easily be collected in large numbers of both cases and controls [14].

Another limitation of the AF approach is that it does not inform on the etiology at an individual level; ie, we do not know which of the test-positive cases have “false-positive” measurements that merely represent colonization, upper respiratory infection, or asymptomatic shedding after an acute infection, rather than the cause of the pneumonia. The option for estimating etiology for an individual case is to assign the group etiologic fraction for pathogens detected in that case, regardless of the findings for other pathogens detected. For example, a case that tested positive for pneumococcus as well as 3 other pathogens will be assigned the same probability of having pneumococcal pneumonia as a case that tested positive for pneumococcus only. Likewise, all children testing negative for pneumococcus will be assigned zero probability of pneumococcal pneumonia. Because attributable fraction does not estimate etiology at the individual case level, it is not possible to characterize the clinical, demographic, radiographic, laboratory, and mortality findings of cases likely attributed to certain pathogens.

Some of these limitations, as well as the different etiologic inferences resulting from different analytic approaches, are illustrated in Table 3 and Figure 1, methods C, D, and E. Case 5 in the line list in Table 3 illustrates the challenge of interpreting etiology using AFE for cases with multiple positive NP PCR measurements significantly associated with case status.

**Table 3.** Integration of Hypothetical Individual Test Results From Pneumonia Cases With Blood Culture and Nasopharyngeal (NP) Polymerase Chain Reaction (PCR) Results, Accounting for Imperfect Specificity of NP PCR

Case	Blood Culture	NP PCR	NP PCR After Case- Control Analysis^a^	NP PCR After AFE Analysis^b^	Integration of Blood Culture and NP PCR
1	*Streptococcus pneumoniae*	**RSV**	RSV	RSV*0.91	*S. pneumoniae* and RSV*0.91
2	*Staphylococcus aureus*	**Influenza A**	Influenza A	Influenza A*0.71	*S. aureus* and influenza A*0.71
3	*Haemophilus influenzae*	**HMPV**	HMPV	HMPV*0.50	*H. influenzae* and HMPV*0.50
4	*Escherichia coli* and *Klebsiella pneumoniae*	Human bocavirus, rhinovirus	Negative	NA	*E. coli* and *K. pneumoniae*
5	Negative	**Influenza A, RSV**	Influenza A, RSV	Influenza A*0.71, RSV*0.91	Influenza A*0.71, RSV*0.91
6	Negative	Human bocavirus, rhinovirus	Negative	NA	Negative
7	Negative	Influenza B, **RSV**	RSV	RSV*0.91	RSV*0.91
8	Negative	Negative	Negative	NA	Negative

Abbreviations: HMPV, human metapneumovirus A/B; NA, not applicable; NP, nasopharyngeal; PCR, polymerase chain reaction; RSV, respiratory syncytial virus A/B.

^a^Pathogens not significantly associated with case status (non–bold text; based on analysis presented in Table 2) are removed from consideration as a cause.

^b^For each pathogen with an odds ratio >1.0 in the case-control analysis, the attributable fraction in the exposed from Table 2 is shown as a multiplier, which is interpreted at the individual level as the probability that the pneumonia is attributed to that pathogen without considering the other test results for that child.

### Approach to Challenge 2. C: Quantification of Pathogen Load

In the field of clinical microbiology, microbes detected in higher quantities are regarded as being more likely to be clinically significant. An association between nasopharyngeal bacterial load and childhood pneumonia has been suggested, and pathogen density in respiratory specimens has been assessed as a measure to distinguish a pathogen as being causative of pneumonia [18–21]. Higher mean or median density of pneumococcal DNA in the upper respiratory tract has been associated with pneumococcal pneumonia in HIV-infected South African adults [18] and radiologically confirmed pneumonia in Vietnamese children [19]. Higher median nasopharyngeal RSV or influenza virus density was found in those with lower respiratory tract infection compared with asymptomatic controls [20]. Despite these findings, most pneumonia studies have found substantial overlap in the density distribution of patients with pneumonia and those with milder or no illness [19, 20, 22, 23]. To date, no clear thresholds in pathogen density distinguish a pathogen as being causative of pneumonia; larger studies or more sophisticated analytical methods may reveal greater interpretive value of density for some pathogens. Additionally, while applying a threshold may improve the specificity, it would result in a loss of sensitivity that would need to be quantified and accounted for.

## Challenge 3: Etiology Due to Pathogens Not Tested For

Pneumonia may be caused by pathogens for which no testing was performed. How much this “none of the above” category contributes to the etiology assessment depends on how many pathogens were tested for, the amount of evidence for those pathogens, and the sensitivity of their diagnostic measurements.

### Approach to Challenge 3: Assign an “Unknown” Etiology

Most often, the proportion of cases with negative results for all tested pathogens is assigned as “unknown” etiology or, if an AF analysis is performed and the sum of the etiologies is <100%, the remaining etiologic fraction is attributed to “unknown” etiology. Because neither of these approaches accounts for the imperfect diagnostic sensitivity of the tests, the cases categorized as “unknown” etiology would reflect cases with false-negative test results, as well as those with a pneumonia caused by pathogens not tested for. For example, the negative measurements for case 8 in Table 3 could be consistent with a false-negative test result for pneumonia caused by a pathogen that was tested for, or with pneumonia caused by a pathogen not tested for, or with a diagnosis other than pneumonia. An approach that also accounts for measurement error would ensure that false-negative results did not contribute to the fraction of cases with “unknown” etiology.

## Challenge 4: Combining Multiple Test Results

Combining results from >1 test or specimen type while simultaneously accounting for the sensitivity and specificity of each one poses an insurmountable challenge when estimating pneumonia etiology using the various methods described above. This is because the analytic methods adjust for measurement error for only one test at a time and only at the population level (ie, for the set of all cases). For cases that are test-positive for >1 pathogen, we cannot determine whether one or both tests are false positive or if the case truly has pneumonia due to multiple etiologies. Consequently, we cannot estimate multiple etiologies at a population level.

This challenge can be illustrated by trying to combine hypothetical blood culture results and nasopharyngeal PCR results for 7 respiratory viruses (Figure 1, methods F, G, and H). Most pneumonia cases have positive viral nasopharyngeal PCR results, with a smaller proportion being positive for bacteria by blood culture, yielding a large number of mixed infections with a variety of pathogen combinations. Testing additional specimens (eg, induced sputum, serum) or targeting more pathogens would result in even more combinations. When using only case results, assuming 100% specificity and without attempting to adjust for diagnostic sensitivity of assays, combining bacterial blood culture and viral nasopharyngeal PCR results is not technically difficult. However, displaying all of the pathogen combinations in a single graphical pie that describes the etiology of pneumonia is visually incomprehensible. For simplicity, results can be categorized as bacterial (cases with only single or multiple bacteria found on blood culture), viral (cases with only single or multiple viruses detected by nasopharyngeal PCR), or mixed bacterial–viral infections (cases with bacteria detected in blood culture and 1 or more viruses detected by nasopharyngeal PCR).

The real challenge of combining results of multiple tests becomes apparent when attempting to account for the poor sensitivity of blood culture and the poor specificity of nasopharyngeal PCR measurements. It is not possible to simultaneously adjust for measurement error and integrate these adjusted measurements. For example, a child with a negative blood culture who tested positive for HMPV could have pneumonia caused by HMPV alone (if the blood culture was a true negative), both HMPV and bacteria (if the blood culture was a false negative, but would not know which bacterial species), bacteria only (if the HMPV was a false positive and the blood culture was a false negative; again, would not know which bacterial species), or something else entirely (true-negative blood culture and false-positive HPMV). Similarly, for cases with multiple positive results, we do not know if the pneumonia was caused by one of these pathogens, multiple pathogens, or other something else entirely (eg, cases 1–5, Table 3). For example, if a case tests positive for RSV and influenza A virus from nasopharyngeal PCR, with AFEs of 0.91 and 0.71, respectively, we cannot determine whether this particular child’s illness is caused by neither, one or both of these pathogens. This challenge becomes increasingly complex with the incorporation of additional pathogens, diagnostic tests and specimens.

### Approach to Challenge 4. A: Expert Adjudication

Analytic integration of multiple positive results has been done using expert adjudication. Such adjudication can use computer algorithms programmed to follow rules set out by the researcher, or it can involve expert review of individual cases with standard clinical principles guiding the adjudication. This is similar to the approach clinicians take when caring for patients, but is not ideal for epidemiologic studies because it is time intensive, does not easily allow for quantification of uncertainty, and is subject to bias. Experts have preconceptions about causes of pneumonia and limited capacity for integrating multiple sources of information on etiology while also robustly accounting for measurement error in detecting causes of pneumonia.

### Approach to Challenge 4. B: Latent Class Analysis

Latent class analysis (LCA) is a case-only statistical approach used to evaluate etiology when multiple measurements for a given pathogen are available, such as pneumococcus detected by blood culture, NP PCR, sputum culture, and lung aspirate culture [24, 25]. The “latent class” refers to the underlying true cause of the child’s pneumonia.

With LCA, cases are grouped into etiologic classes (the number of which must be prespecified by the researcher) based on their pattern of measurements, with cases in a class being more similar to one another with respect to those measurement patterns than cases in other classes. The measurements usually include a variety of imperfectly sensitive or specific tests (eg, culture of blood, PCR of upper respiratory tract specimens). A key assumption is that the measurements are independent of one another within a class. For each class, the analysis estimates the probability distribution of each measurement (eg, proportion blood culture positive for pneumococcus within that class) and the proportion of cases attributed to that class. At the individual level, cases have a probability of belonging to each class (eg, 20% in class A and 80% in class B).

LCA is best used to distribute cases into a limited number of clearly defined classes, such as the probability of being caused by *Streptococcus pneumoniae* or not. Because of computational limitations, LCA is not an option for evaluating large numbers of pathogens. LCA also does not determine the etiology for each class. Rather, the researcher must “interpret” the measurement distribution profiles of each class and ascribe a pathogen or a combination of pathogens as the etiology for each class.

## Challenge 5: A Comprehensive Approach to Address All Challenges Above

The limitations of the existing analytic methods for etiologic analyses pose more of a challenge for pneumonia than for some other illnesses for several reasons. Because specimens are rarely available from the lung, multiple specimens from sites peripheral to the pneumonia infection are often tested, each contributing different kinds of information; only a subset of specimens are available from nonpneumonia control subjects. Often many pathogens are detected in the specimens (including specimens from controls without pneumonia) and some pathogens have more types of tests available than others. Depending on the specimens collected, pathogens targeted for detection, and analytic methods used, substantially different conclusions are drawn about pneumonia etiology between studies (Figure 1).

### Approach to Challenge 5: Partial Latent Class Analysis

To address the limitations of existing analytic methods, the Pneumonia Etiology Research for Child Health (PERCH) study—a case-control study of pediatric pneumonia etiology in 7 African and Asian countries—has developed a partial latent class, Bayesian approach (pLCA), to estimate pneumonia etiology at the individual and population levels [26–28].

The pLCA approach can integrate multiple types of measurements, account for their respective sensitivity and specificity (by incorporating data from controls) at the pathogen level, accommodate a large number of pathogens, and estimate the proportion of cases with an etiology due to pathogens not tested for. This method, while not solving all of the fundamental design issues of pneumonia etiology studies (eg, indirect observation of infection site, not all specimen types available from controls) and introducing other challenges (eg, the need for a prespecified prior distribution for key parameters), addresses many of the limitations of the analytic methods described above. The approach can evaluate evidence for multiple-pathogen infections; estimating the likelihood of a given case of pneumonia being caused by a single or multiple pathogens will be part of a future version of the pLCA.

## CONCLUSIONS

Determining pneumonia etiology poses numerous analytical challenges. The imperfect diagnostic sensitivity and specificity of laboratory tests require that epidemiologists and researchers view laboratory results with a more critical eye, rather than taking them at face value, and explore new interpretive approaches. More advanced analytic techniques for cross-sectional data, such as the pLCA approach developed for the PERCH study, address many of the limitations of earlier approaches. However, even the pLCA approach cannot overcome some of the fundamental limitations of the current state of pneumonia etiology research: tests have measurement error, the ideal number of pathogens to test for is unknown, and most importantly, the specimens used to determine pneumonia etiology are generally not from the lung. Ultimately, a better understanding of pneumonia etiology will likely come from using advanced, integrated analytic techniques, such as pLCA, on cross-sectional pneumonia studies, complemented by other designs that employ different angles of approach to the etiology question, such as longitudinal cohorts, vaccine probes, and postmortem and lung aspirate studies.

## Supplementary Data

Supplementary materials are available at *Clinical Infectious Diseases* online. Consisting of data provided by the author to benefit the reader, the posted materials are not copyedited and are the sole responsibility of the author, so questions or comments should be addressed to the author.

## Supplementary Material

OTH1_SupplementalMaterial

## Notes

***Author contributions.*** L. L. H. and M. D. K. drafted the initial manuscript. D. R. F., J. A. G. S., S. L. Z., D. R. M., and K. L. O. provided significant contributions to the manuscript. All authors reviewed and approved the manuscript. L. L. H. had ﬁnal responsibility for the decision to submit for publication.

***Acknowledgments.*** We are grateful to the PERCH Study Group members who contributed to the study design, conduct, and/or analysis phases of PERCH (see Supplementary Materials for full list of names). We also acknowledge Zhenke Wu and Mingfan Pan for their support with the analyses.

***Financial support.*** PERCH was supported by the Bill & Melinda Gates Foundation (grant number 48968 to the International Vaccine Access Center, Department of International Health, Johns Hopkins Bloomberg School of Public Health). J. A. G. S. was supported by a clinical fellowship from The Wellcome Trust of Great Britain (award number 098532).

***Supplement sponsorship.*** This article appears as part of the supplement “Pneumonia Etiology Research for Child Health (PERCH): Foundational Basis for the Primary Etiology Results,” sponsored by a grant from the Bill & Melinda Gates Foundation to the PERCH study of Johns Hopkins Bloomberg School of Public Health, Baltimore, Maryland.

***Potential conﬂicts of interest.*** M. D. K. has received funding for consultancies from Merck, Pfizer, and Novartis, and grant funding from Merck. L. L. H. has received grant funding from Pfizer and GlaxoSmithKline. K. L. O. has received grant funding from GSK and Pfizer and participates on technical advisory boards for Merck, Sanofi Pasteur, PATH, Affinivax, and ClearPath. All other authors report no potential conflicts. All authors have submitted the ICMJE Form for Disclosure of Potential Conflicts of Interest. Conflicts that the editors consider relevant to the content of the manuscript have been disclosed.
